# Supplementary material for: A Simple Reversed Iontophoresis-Based Sensor to Enable In Vivo Multiplexed Measurement of Plant Biomarkers Using Screen-Printed Electrodes
Source: Sensors (Basel). 2023 Jan 10;23(2):780. doi: 10.3390/s23020780 (PMC9863583; doi:10.3390/s23020780)
Supplement: Supplementary file 1 [file sensors-23-00780-s001.zip › sensors-2092836-supplementary.pdf]

Supplementary Figures

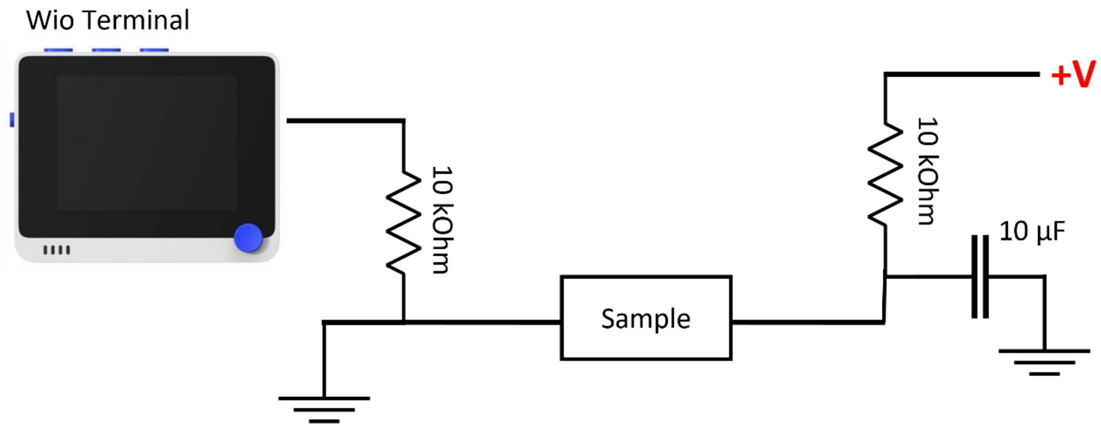

Figure S1. Circuit employed for the voltage application and current measurement during the reverse iontophoresis process. A 10 kOhm resistor was used as a reference, allowing an estimation of the employed current.

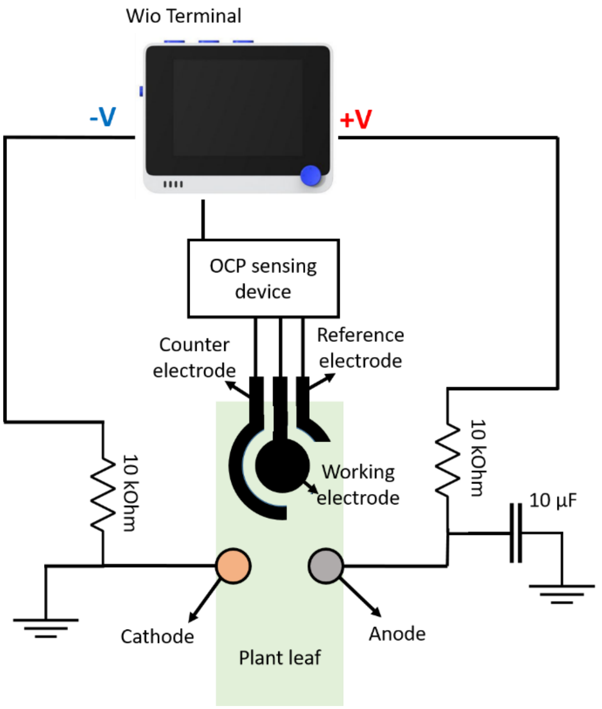

Figure S2. Schematic representation of the full electronic circuit used in the present work, including the cathode and anode connections used in the extraction of ions from plant leaves, and pH quantification, as well as the screen-printed electrodes used in the measurement of K^+.

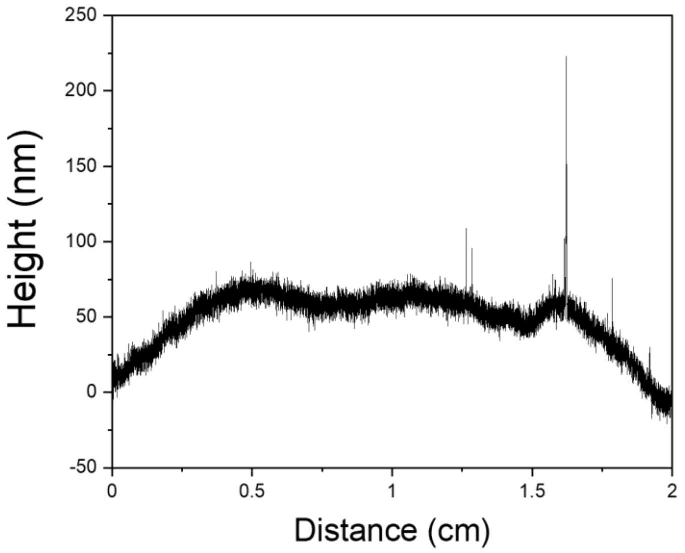

Figure S3. Height profile of PVC-based ion-selective electrode obtained using stylus profilometer.

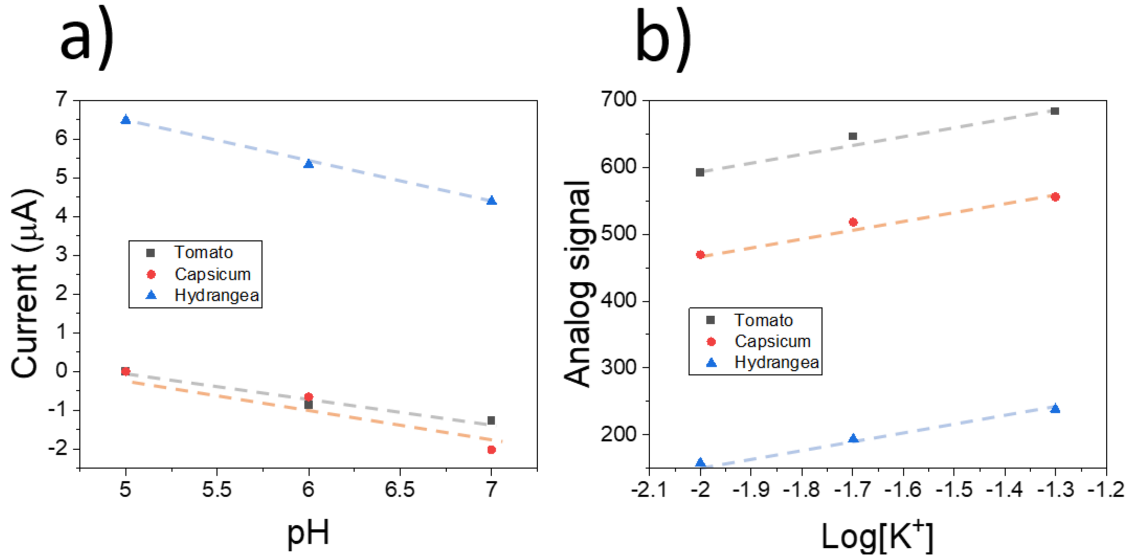

Figure S4. a) Calibration plot of sensing devices placed onto different plant leaves, evidencing the robustness of the sensing method. **b)** Calibration plot obtained in the case of the K^+ concentration.

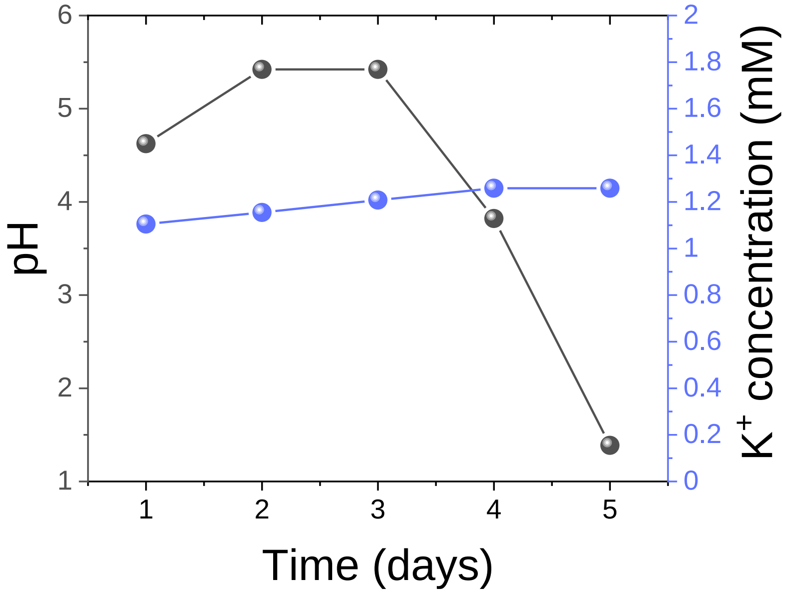

Figure S5. Plot evidencing the stability of the sensing devices. Sensors were tested daily, and the values of pH and K^+ concentrations were measured. A significant decrease in pH measurements was recorded after 3 days of operation, due to a degradation of RuO_x films.
